# L (+)-lactic acid production by pellet-form *Rhizopus oryzae NRRL 395* on biodiesel crude glycerol

**DOI:** 10.1186/1475-2859-12-92

**Published:** 2013-10-10

**Authors:** Dan C Vodnar, Francisc V Dulf, Oana L Pop, Carmen Socaciu

**Affiliations:** 1 Food Science and Technology Department, Unit of Chemistry and Biochemistry, University of Agricultural Sciences and Veterinary Medicine, 3-5 Mănăştur str, Cluj-Napoca 400372, România; 2 Department of Environmental and Plant Protection, University of Agricultural Sciences and Veterinary Medicine, 3-5 Mănăştur str, Cluj-Napoca 400372, România

## Abstract

**Background:**

Given its availability and low price, glycerol derived from biodiesel industry has become an ideal feedstock for the production of fuels and chemicals. A solution to reduce the negative environmental problems and the cost of biodiesel is to use crude glycerol as carbon source for microbial growth media in order to produce valuable organic chemicals. In the present paper, crude glycerol was used as carbon substrate for production of L (+)-lactic acid using pelletized fungus *R. oryzae NRRL 395* on batch fermentation. More, the experiments were conducted on media supplemented with inorganic nutrients and lucerne green juice.

**Results:**

Crude and pure glycerols were first used to produce the highest biomass yield of *R. oryzae NRRL 395*. An enhanced lactic acid production then followed up using fed-batch fermentation with crude glycerol, inorganic nutrients and lucerne green juice. The optimal crude glycerol concentration for cultivating *R. oryzae NRRL 395* was 75 g l^-1^, which resulted in a fungal biomass yield of 0.72 g g^-1^ in trial without lucerne green juice addition and 0.83 g g^-1^ in trial with lucerne green juice. The glycerol consumption rate was 1.04 g l^-1^ h^-1^ after 48 h in trial with crude glycerol 75 g l^-1^ while in trial with crude glycerol 10 g l^-1^ the lowest rate of 0.12 g l^-1^ h^-1^ was registered. The highest L (+)-lactic acid yield (3.72 g g^-1^) was obtained at the crude glycerol concentration of 75 g l^-1^ and LGJ 25 g l^-1^, and the concentration of lactic acid was approximately 48 g l^-1^.

**Conclusions:**

This work introduced sustainable opportunities for L (+)-lactic acid production via *R. oryzae NRRL 395* fermentation on biodiesel crude glycerol media. The results showed good fungal growth on crude glycerol at 75 g l^-1^ concentration with lucerne green juice supplementation of 25 g l^-1^. Lucerne green juice provided a good source of nutrients for crude glycerol fermentation, without needs for supplementation with inorganic nutrients. Crude glycerol and lucerne green juice ratio influence the L (+)-lactic acid production, increasing the lactate productivity with the concentration of crude glycerol.

## Background

Energy fuels for world consume are mainly derived for finite and declining reserves of fossil hydrocarbons [1]. Now, there is a global dependency on fossil hydrocarbons, which will not be environmentally and economically sustainable in the long term [1]. Nowadays, having the authority’s pessimistic prospects for the future, regarding the absolute dependency on fossil fuels, the political and economical policies are to stimulate the development of sustainable alternatives to fossil fuels. Biodiesel, represent an alternative for the substitution of fossil fuels, which is the most common biofuel in Europe [2]. Similar to the petroleum industry, biodiesel industry generate unwanted by-products [1]. Biodiesel production generate huge amount of crude glycerol- one part of glycerol is produced at every ten parts of biodiesel [3] and has a negative influence on the biodiesel price. The glycerine phase from biodiesel industry causing the environmental problems regarding to the management of this by-product. A solution to reduce the negative environmental problems and the cost of biodiesel is to use crude glycerol as carbon source for microbial growth media in order to produce valuable organic chemicals [4,5].

Lactic acid (lactate) and its derivates have many applications in the food, pharmaceutical, and polymer industries [6]. Its most promising application is in being used as a major raw material for the production of polylactic acid (PLA) [7]. In this context, lactic acid can be produced via biological route having the advantage of being able to produce optically pure lactic acid, while chemical route produce racemic mixture with the requirements of high temperature and pressure [8,9].

Lactic acid from biological sources can be produced by both bacteria and fungi fermentation [10]. Production of L (+)-lactic acid via fermentative route has increased in the last decade [7]. For the industrial production of L (+)-lactic acid, it is necessary to provide cheap carbon sources, and to obtain the optimal conditions of fermentation with higher yields and production rates [11]. Generally, bacterial fermentation has a higher yield [12]. Apart from the large group of bacteria, filamentous fungi, especially *R. oryzae* have been proving a good lactic acid producer. *R. oryzae NRRL 395* can utilize crude glycerol as carbon source, and unlike its competitors of lactic acid producing bacteria, tolerate high impurities, has lower nutrition requirements which reduce the fermentation cost and simplifies downstream product separation [13,14] it is more tolerant to a low pH environment and the fungal biomass is easy to separate from broth [10].

In the lactic acid production by *R. oryzae NRRL 395*, a considerable amount of chitin is produced [10], which limited the mass transfer of oxygen and nutrients onto the fungus and the release of the organic acid media. Growing fungi in pellet form can reduce these problems.

The aim of the current investigation was to further assess the potentialities of valorization of crude glycerol by pelletized *R. oryzae NRRL 395*, in order to produce L (+)-lactic acid in media supplemented with laboratory nutrients or with lucerne green juice. The pelletized fermentation hold great promise as potential bioconversion route of low-value glycerol streams to a higher-value product like lactic acid.

## Results and discussion

### *R. oryzae NRRL 395* biomass production

**Figure 1:**
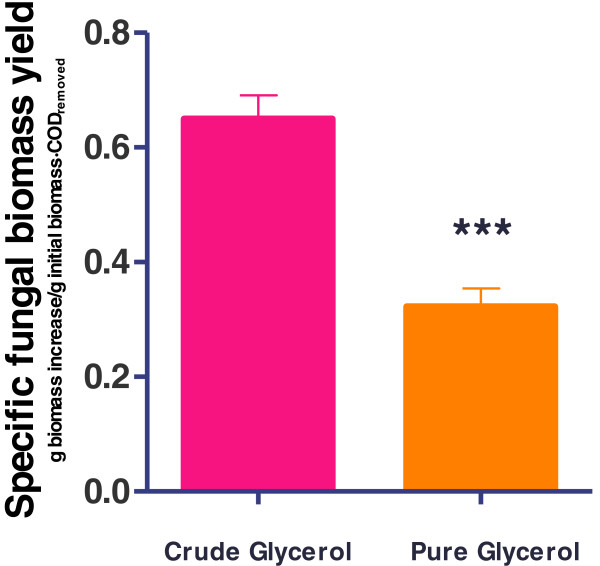
**Specific fungal biomass yield for samples with pure glycerol and crude glycerol (30 g l**^**-1**^**) supplemented with inorganic nutrients (IN).** The bars are means of three determinations ± SD. *** Extremly significant according to T-test.

*R. oryzae NRRL 395* was successfully cultivated on various biofuel residues, but the extremely high organic content of the crude glycerol was a major concern prior to start this work. Thus, the first study was conducted to evaluate the possibility of *R. oryzae NRRL 395* to use the crude glycerol as sole carbon source. Nutrient supplemented media containing different carbon sources 30 g l^-1^ including, pure glycerol and crude glycerol were selected. The initial organic matters (COD) were different in the substrates. The fungal biomass yield was used to evaluate the feasibility of *R. oryzae NRRL 395* cultivation on these media. A statistical analysis showed significant differences between the specific fungal biomass yields on crude glycerol and pure glycerol samples at 95% confidence (Figure 1). The highest specific biomass yield of 0.65 ± 0.02 g biomass increase/g initial biomass · g COD_removed_) was obtained on crude glycerol samples. This demonstrated that *R. oryzae NRRL 395* was able to use crude glycerol as carbon source. These results were in agreement with those reported by Nitayavardhana et al. [15], in which *R. microsporus* var. *oligosporus* showed a specific biomass yield of 0.64 ± 0.06 g on crude glycerol. More, the work reported insignificant differences between fungal biomass yields on media with yeast extract and crude glycerol.

**Figure 2:**
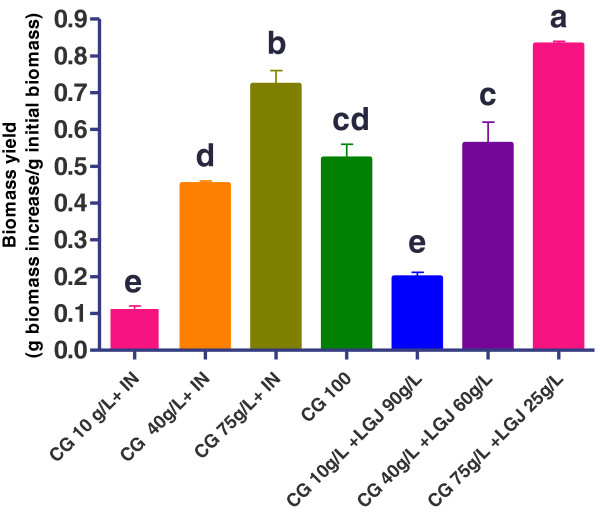
***R. oryzae NRRL 395 *biomass yield (g biomass increase/g initial biomass) on media containing different ratio of crude glycerol (CG) (10, 40, 75, 100 g l**^**-1**^**) supplemented with inorganic nutrients (IN) or with lucerne green juice (LGJ 90, 60, 25 g l**^**-1**^**).** Means (n = 3) ± SD. Means with different letter are significantly different (p < 0.05).

As shown in Figure 2, the optimal crude glycerol concentration for cultivating *R. oryzae NRRL 395* was 75 g l^-1^, which resulted in a fungal biomass yield of 0.72 g g^-1^ in trial without LGJ addition and 0.83 g g^-1^ in trial with LGJ. With respect to the control (CG 100 g l^-1^, without nutrient supplementation), at the optimal glycerol concentration (75 g l^-1^) and pH 5, an improvement in fungal biomass yield was observed with 0.2 g biomass when crude glycerol samples were supplemented with inorganic nutrients. These results were in agreement whit those reported by Nitayavardhana and Khanal, [16] which demonstrated over 200% increase in fungal biomass yield in vinasse media supplemented with nitrogen and phosphorus. Generally, the fungal biomass was lower in crude glycerol media supplemented with nutrients than in vinasse. However, nutrient supplements had a lower solubility on crude glycerol, thus led to a slower fungal growth and the use of low-cost nutrient-rich solution will overcome this problem [16].

Another study shows that Lucerne green juice can be use as inexpensive nutrient in lactic acid fermentation [17]. The extracted juice was successfully used as substitute for expensive nutrients for cultivating *Lactobacillus paracasei 168*. The study noticed that partial substitution of expensive media components was possible by using lucerne green juice. This finding suggested that lucerne green juice constituted a nutrient supplement for crude glycerol fermentation, substituting the laboratory expensive nutrients used for this kind of fermentations. The lucerne green juice characteristics are summarized in Table 1. It is interesting to note that fungal growth in samples with crude glycerol 10 g l^-1^ and 90 g l^-1^ LGJ was not significantly different compared with crude glycerol 10 g l^-1^ supplemented with laboratory inorganic nutrients. As shown in Figure 2, a significant fungal biomass yield was observed in crude glycerol supplemented with lucerne green juice LGJ 60 g l^-1^ (0.83 g biomass) and LGJ 25 g l^-1^ (0.52 g biomass). This supports the potential of utilizing a low-cost lucerne green juice as a source of nutrients to enhance the growth of *R. oryzae NRRL 395*.

**Table 1:** Characteristics of lucerne green juice

**Parameters**	Values*
pH	5.98 ± 0.1
Sugar [g/L]	16.8 ± 0.15
N_tot_ [g/L]	4.5 ± 0.11
P_tot_ [g/L]	3.47 ± 0.11
DM [%]	6.12 ± 0.19
NO_2_^–^ [mg/L]	15.65 ± 1.62
NO_3_^–^ [mg/L]	7.50 ± 0.85
Cl^–^ [mg/L]	1560 ± 64
SO_4_^2–^ [mg/L]	596 ± 36
PO_4_^3–^ [mg/L]	556.3 ± 24.16
Na^+^ [mg/L]	81.7 ± 1.29
K^+^ [mg/L]	4968.5 ± 37.27
Mg^2+^ [mg/L]	437.6 ± 16.2
Ca^2+^ [mg/L]	1289 ± 11.3

*All analysis are based on n (sample size) = 3.

### Kinetics study of lactic acid production in bioreactor

#### Lactic acid production by *R. oryzae NRRL 395* on glucose substrate

**Figure 3:**
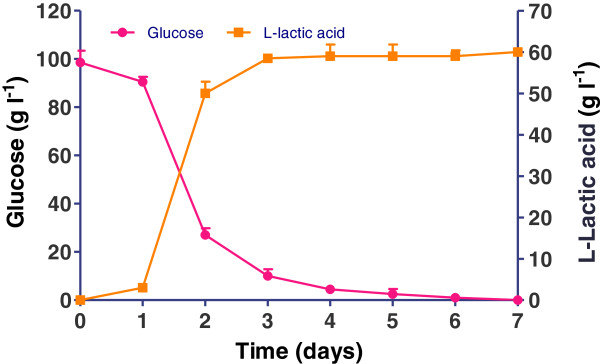
**Substrate consumption and product yield curves of *R. oryzae NRRL 395 *during the fermentation conditions on media with glucose (101 g l**^**-1**^**).** The bars are means of three determinations ± SD.

First, the capacity of *R. oryzae NRRL 395* for sugar utilization was investigated on glucose as model media. The observation of *R. oryzae NRRL 395* in consuming glucose and producing L (+)-lactic acid was presented in Figure 3.

The initial concentration of glucose was 101 g l^-1^. During the first day of growth, the *R. oryzae NRRL 395* was adapted itself to the media conditions without showing substantial changes in concentrations of either glucose or L (+)-lactic acid in the culture medium. In the next 2 days, *R. oryzae NRRL 395* achieved a maximal yield of L (+)-lactic acid after 72 hours of fermentation, indicated by the major consumption of nutrients during this period. In the next 3 days, there was almost no additional L (+)-lactic acid produced (the level remained constant around 59 g l^-1^). The glucose consumption decreased sharply from 90.5 to 27 g l^-1^ after 48 h and all the glucose was largely used up after 72 h. For this experiment setup, 3 days of incubation were sufficient for the system to reach the stage highest concentration (58.41 g l^-1^) of L (+)-lactic acid. These results were in agreement to those reported by Yao et al. [18], in which the final lactic acid concentration of 52 g l^-1^ was achieved after 72 h of fermentation.

### Lactic acid production by *R. oryzae NRRL 395* on crude glycerol supplemented with inorganic nutrients

**Figure 4:**
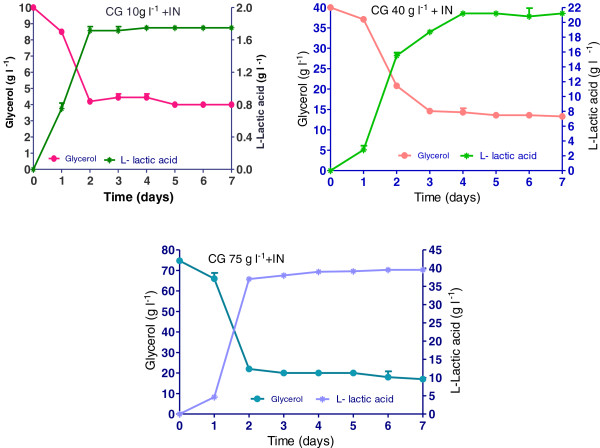
**L (+)-lactic acid production and glycerol consumption obtained in trials with crude glycerol (CG) (10, 40, and 75 g l**^**-1**^**) supplemented with inorganic nutrients.** The bars are means of three determinations ± SD.

**Figure 5:**
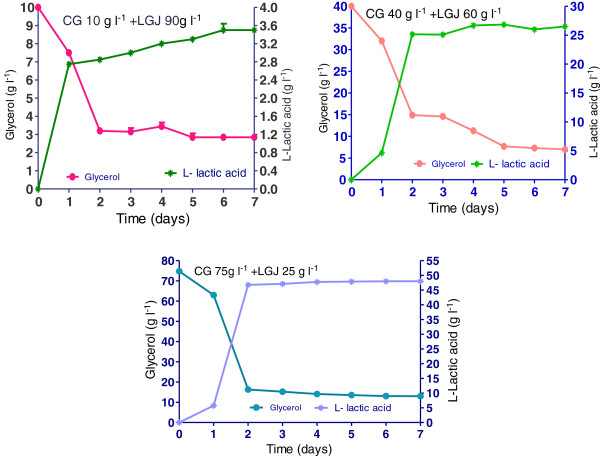
**L (+)-lactic acid yield (g L (+)-lactic acid/g glycerol) during *R. oryzae NRRL 395 *fermentation in media containing different ratio of crude glycerol (CG) (10, 40, and 75 g l**^**-1**^**) supplemented with inorganic nutrients (IN) or with lucerne green juice (LGJ) (90, 60, and 25 g l**^**-1**^**).** The error bars in the figure indicate the standard deviations of three parallel replicates ± SD.

**Figure 6:**
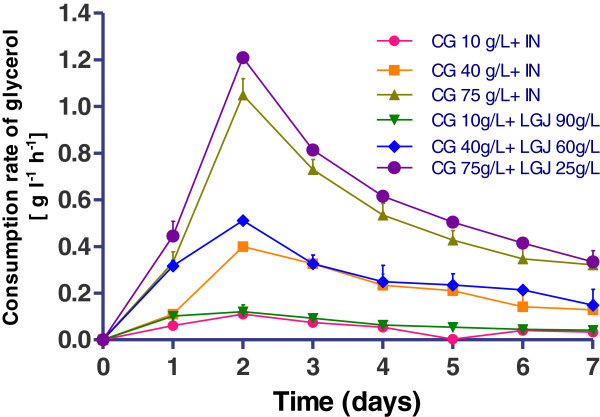
**L (+)-lactic acid productivity [concentration of L (+)-lactic acid (in g l**^**-1**^**) / fermentation time (in h)] during*R. oryzae NRRL 395*fermentation in media containing different ration of crude glycerol (CG) (10, 40, and 75 g l**^**-1**^**) supplemented with inorganic nutrients (IN) or with lucerne green juice (LGJ) (90, 60, and 25 g l**^**-1**^**).** The error bars in the figure indicate the standard deviations of three parallel replicates ± SD.

Figure 4 demonstrated the trends of glycerol consumption and the L (+)-lactic acid formulation in media with crude glycerol (CG), supplemented with inorganic nutrients. *R. oryzae NRRL 395* in the culture media with crude glycerol 10 g l^-1^ consumed 6.1 g l^-1^ glycerol and produced 1.75 g l^-1^ L (+)-lactic acid, showed a lactate yield of 0.34 g g^-1^ (Figure 5) and a productivity of 0.09 g l^-1^ h^-1^ (Figure 5). Increasing the crude glycerol concentration in the media was considered a possibility to reach higher lactic acid accumulation at the end of fermentations. The highest concentration of glycerol (61.5 g l^-1^) used by *R. oryzae NRRL 395* was in fermentation trial with 75 g l^-1^ addition of crude glycerol. In this case the lactate yield (Figure 5) and productivity (Figure 6) was 1.64 g g^-1^ and 0.73 g l^-1^ h^-1^ respectively, after 48 hours, but in a longer fermentation time (168 h) the final lactate yield was 2.31 g g^-1^. In trial with 40 g l^-1^ crude glycerol, the lactate yield was 1.59 g g^-1^ producing 13.3 g l^-1^ lactic acid after 7 days of fermentation. The glycerol consumption rate was 1.04 g l^-1^ h^-1^ after 48 h in trial with crude glycerol 75 g l^-1^ while in trial with crude glycerol 10 g l^-1^ the lowest rate of 0.12 g l^-1^ h^-1^ was registered. Based on this data, was apparent that crude glycerol and inorganic nutrient supplements represented a good source for *R. oryzae NRRL 395* growth and lactic acid production. There are many factors that could influence the growth of *R. oryzae NRRL 395* such as: medium nutrients, pH, agitation, aeration, medium viscosity and inoculum size [19-21]. Some strains such as *Rhizopus.sp*. requires strong agitation to form pellets, while others strains needs high pH [22]. Vially et al. [23] reported no lactate production on glycerol or lactose when *R. oryzae NRRL 395* UMIP 4.77 was used at a stirring rate of 200 rpm. In contrast, Abe et al. [24] founded that *R. oryzae NRRL 395* grew vigorously on glycerol but poorly on sucrose and maltose.

### Lactic acid production by *R. oryzae NRRL 395* on crude glycerol supplemented with lucerne green juice

**Figure 7:**
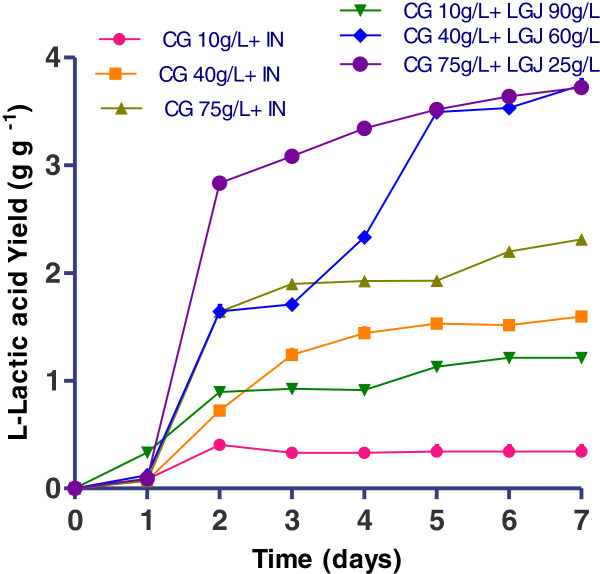
**L (+)-lactic acid production and glycerol consumption obtained in trials with crude glycerol (CG) (10, 40, and75 g l**^**-1**^**) supplemented with lucerne green juice (LGJ) (90, 60, and 25 g l**^**-1**^**).** The bars are means of three determinations ± SD.

**Figure 8:**
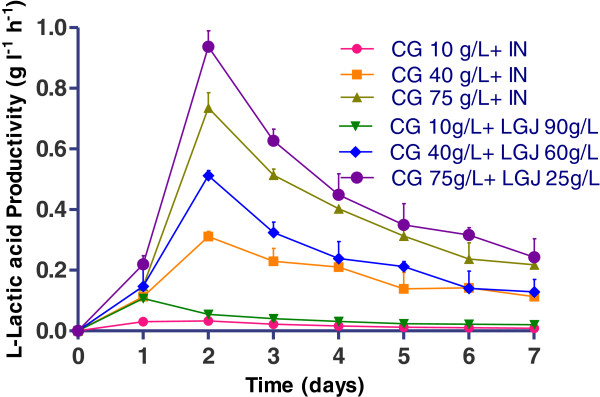
**Consumption rate of glycerol [initial concentration of glycerol (g l**^**-1**^**) - residual concentration of glycerol (g l**^**-1**^**)]/fermentation time [in h], during *R. oryzae NRRL 395 *fermentation in media containing crude glycerol (CG) (10, 40, and 75 g l**^**-1**^**) supplemented with inorganic nutrients (IN) or with lucerne green juice (LGJ) (90, 60, and 25 g l**^**-1**^**).** The error bars in the figure indicate the standard deviations of three parallel replicates ± SD.

Stirred –tank bioreactor experiments showed the *R. oryzae NRRL 395* capacities to grow and produce L (+)-lactic acid on crude glycerol media supplemented with lucerne green juice. As shown in Figure 7 and Figure 8, the highest L (+)-lactic acid yield (3.72 g g^-1^) was obtained at the crude glycerol concentration of 75 g l^-1^ and LGJ 25 g l^-1^, and the concentration of lactic acid was approximately 48 g l^-1^. When the LGJ 90 g l^-1^ was used, the consumption rate (Figure 8) of glycerol registered after 48 hours of fermentation was 0.12 g l^-1^ h^-1^while in trials with 60 g l^-1^ and 25 g l^-1^ LGJ the consumption rate was 0.51 g l^-1^ h^-1^ and 1.2 g l^-1^ h^-1^, respectively. In comparison with glucose (0.58 g g^-1^) (Figure 3) the lactic acid yield was higher in all trials which used LGJ and less in trial with crude glycerol 10 g l^-1^ supplemented with inorganic nutrients (0.34 g g^-1^). Generally, the profile in bioreactor fermentation could be divided into two parts at the time point of 48 h (Figure 7). Prior to 48 h, 46.75 g l^-1^ L (+)-lactic acid was produced in trial with 25 g l^-1^ LGJ addition, with a productivity of 0.93 g l^-1^ h^-1^ which was higher than the rest of the trials. From 48 h to 168 h, L (+)-lactic acid concentration reached at a value higher than 47.1 g l^-1^. At the end of the fermentation 48.1 g l^-1^ L (+)-lactic acid was obtained. The pellet formation observed in these trials might be attributed to the nitrogen and phosphorus content of lucerne green juice that increased the viscosity of culture medium and influenced the *R. oryzae NRRL 395* growth. More, the potassium content and protein content of lucerne green juice might increase the growth rate of *R oryzae* in the culture conditions studied. Because of low price and availability, lucerne green juice was a possible feedstock for lactate production. As the increasing interest in producing biotechnological products from low-cost and renewable biomass, the production of lactic acid from various materials has gained attention recently. Fungal species and lactic acid bacteria (LAB) have been investigated for production of lactic acid. Different yields of L (+)-lactic acid had been reported during fermentation of crude glycerol by LAB strains (0.23-0.71 g g^-1^) [25] or by fungi (0.25-0.86 g g^-1^) [23].

### FTIR characterization

**Figure 9:**
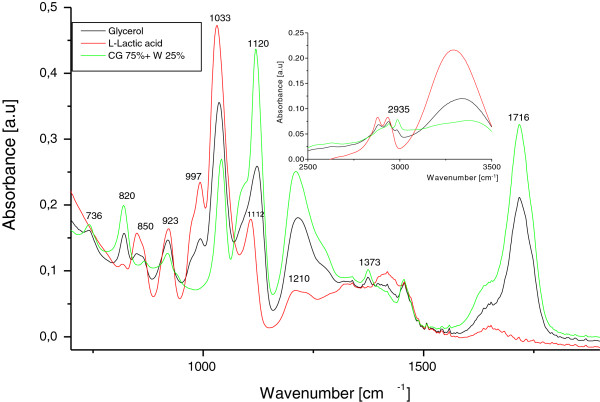
**Comparative FTIR fingerprint of glycerol, lactic acid and the sample at the end of the fermentation process from trial with crude glycerol (CG) (75 g l**^**-1**^**) supplemented with inorganic nutrients (IN).**

FTIR spectroscopy was expected to be especially valuable in analyzing the phase structure and the product formulation during pelletized fungal fermentation. The infrared spectra of lactic acid, crude glycerol and the sample at the end of the fermentation process from trial with crude glycerol 75 g l^-1^ supplemented with inorganic nutrients were showed in Figure 9. The functional group of glycerol, including O-H stretching at 3312 cm^-1^, C-H stretching at 2983, 2935, 2883 cm^-1^, C-O stretching from 1100 cm^-1^ as primary alcohol to 1400 cm^-1^ represented the secondary alcohol, C-C and C-O stretching from 995 to 1050 cm^-1^[26]. Specific peak for lactic acid was located at 1112 cm^-1^[27]. To study the lactic acid formulation, we followed the band corresponding to lactic acid, which showed changes in the spectra during fermentation processes. These signals validated the HPLC investigations regarding the concentration of lactic acid.

## Conclusions

This work introduced sustainable opportunities for L (+)-lactic acid production via *R. oryzae NRRL 395* fermentation on biodiesel crude glycerol media. The results showed good fungal growth on crude glycerol at 75 g l^-1^ concentration with lucerne green juice supplementation. Lucerne green juice provided a good source of nutrients for crude glycerol fermentation, without required for supplementation with inorganic nutrients. Crude glycerol and lucerne green juice ratio influenced the L (+)-lactic acid production, increased the lactate productivity once with the concentration of crude glycerol. Further research on scaling –up the process and monitorization by FTIR spectroscopy of L (+)-lactic acid production on bioreactor is required.

## Methods

### Glycerol

Crude glycerol was derived from the transesterification reaction of soybean oil and ethanol catalyzed by NaOH. The pH of glycerine phase was corrected with H_2_SO_4_ (1 N) to eliminate the free alkalinity. It was then subject to heating (up to 120°C for approximatively 1 h) under agitation to eliminate ethanol. The sulphate resulting from the neutralization was separated by decantation during 24 h.

### Lucerne green juice

From the pressing procedure, 2.13 kg green juice and 2.87 kg press cake were obtained from 5 kg chopped Lucerne. The juice was drawn off into plastic containers stored until required, at temperature of -18°C. The important characteristics of lucerne green juice are summarised in Table 1.

### *R. oryzae NRRL 395* cultivation

The fungus *R. oryzae NRRL 395* was obtained from USAMV microorganism bank. The fungus was first grown on potato-dextrose agar (PDA) (Sifin) slants at 30°C for 7 days. For experiments, the fungal spores in the slant were resuspended in sterilized water maintaining at 3°C. In terms of achieving pellet form, the spore solution was inoculated in a 125 ml Erlenmeyer flask, containing 50 ml of seed medium with a spore concentration of 1 x 10^7^spore/ml, and cultured at 27°C on shaker (Heidolph Unimax 1100) set at 180 rot/min for 24 hours. The cultured temperature was set at 27°C. The pellet was decanted by centrifugation at 15000 rpm for 15 min and used for the following experiments.

### Kinetic study of lactic acid production in bioreactor

The kinetic study was performed on media containing 10 g l^-1^, 40 g l^-1^, 75 g l^-1^ and 100 g l^-1^ crude glycerol with tap water and inorganic nutrient supplementation. Media with inorganic nutrient supplementation contained 0.0092% CaCl_2_ · 2H_2_O, 0.4% MgSO_4_ · 7H_2_O, 0.6% KH_2_PO_4_, and 1.6% (NH_4_)2SO_4_. The second scenario consisted in the substitution of inorganic nutrient supplements with lucerne green juice, thus the fermentation media containing 10 g l^-1^, 40 g l^-1^, 75 g l^-1^ crude glycerol with lucerne green juice 90 g l^-1^, 60 g l^-1^, and 25 g l^-1^. In all samples the pH was adjusted to 5.5 with calcium carbonate (35 g l^-1^). The bioreactor containing the medium was sterilized in autoclave at 121°C for 15 min. Inoculum was transferred to the fermentation media at 1 x 10^7^spore/ml. The bioreactor used for the fermentative assays was the Electrolab FERMAC 360 with a vessel volume of 2.9 L, equipped with a digital control unit. The fermentation was carried out at 27°C with 360 rpm agitation speed and 1 vvm aeration rate.

### Analytical methods

The lucerne green juice samples were dried to a constant weight at 102°C to dry matter (DM102). For total solids analysis, the dried residue was weighed and heated in a crucible for 2 h at 550°C in a preheated furnace. After cooling, the crucible and ash were weighed. The total nitrogen content (N_tot_) was analyzed using the standard method *Kjeldahl*. The colorimetric technique was used to measure the total phosphorus with the molybdenum blue method. Anions and cations were determined under the following conditions using the ion chromatograph Agilent 1200 Series HPLC: IonPac AS14 (4 mm) column (anions) and IonPac CS12A (4 mm) column (cations). The eluent was 3 mM disodium carbonate, 1 mM sodium hydrogen carbonate (anions) and 23 mM sulfuric acid (cations) with a flow rate of 1 mL/min. The injection volume was 20 μL. Aliquots of the fermentation liquid were taken every 24 h to determine L-lactic acid and glycerol concentration. The content of lactic acid and glycerol was determined by HPLC (Dionex) using a Eurokat H column (300 × 8 mm, 10 lm) and an differential refractometer RI-71 detector with a detection limit of 0.01 g/L. The mobile phase was 0.01 NH_2_SO_4_ using isocratic elution with a flow rate of 0.9 mL/min. The chemical oxygen demand (COD) was determined by Standard Methods #5220 [28].

The yield of L (+)-lactic acid, productivity and the consumption rate of glycerol were calculated as:

$$\text{Yield}\;\text{of}\;\text{lactic}\;\text{acid}=g\;L\;\left(+\right)‒\text{lactic}\;\text{acid}/g\;\text{glycerol}$$

$$L\;\left(+\right)-\text{lactic}\;\text{acid}\;\text{productivity}=\text{concentration}\;\text{of}\;L\;\left(+\right)‒\text{lactic}\;\text{acid}\;\left(\text{in}\;g\;1^{‒1}\right)/\text{fermentation}\;\text{time}\;\left(\text{in}\;h\right)$$

$$\begin{array}{l} \text{Consumption}\;\text{rate}\;\text{of}\;\text{glycerol}=\text{initial}\;\text{concentration}\;\text{of}\;\text{glycerol}\;\left({g\;l}^{-1}\right)\\ \;-\text{residual}\;\text{concentration}\;\text{of}\;\text{glycerol}\;\left({g\;l}^{-1}\right)/\text{fermentation}\;\text{time}\;\left(\text{in}\;h\right) \end{array}$$

### Biomass yield and specific biomass yield

The biomass yield was determined following 96 hours of fermentation. The inoculum for trials was fixed at 0.12 g dry pellet biomass l^-1^. The collected samples after the fermentation were centrifuged at 4000 rpm for 15 min. The precipitated biomass was washed twice using 4 N HCl to remove residual calcium carbonate and the biomass was determined by weighing the mass after drying at 80°C overnight.

Biomassyield=gbiomassincrease/ginitialbiomass

*Specific biomass yield* = grams biomass increase/gram initial biomass· COD removed, and was determined in order to compare fungal growth in various samples with different initial organic content.

### FTIR characterization of the fermentation processes

FTIR spectra using attenuated total reflectance (ATR) and an internal reflection accessory made of composite zinc selenide (ZnSe) and diamond crystals were obtained on a Schimatzu IR Prestige- 21 spectrometer. Each spectrum was registered from 4000 to 500 cm^-1^. The FTIR spectra were recorded for all samples in parallel with controls. Three spectra were acquired for each trial variant at room temperature. Each spectrum was composed of an average of 128 separate scans. The measuring time was approximately 9 minutes per sample (n = 3), depending on the number of scans per spectrum. Accordingly, as the average number of scans increased, the measuring time increased.

### Statistical analysis

All experiments were conducted in triplicates and ANOVA and t-tests were employed to determine if there was statistical difference between trials at significance level of p < 0.05 using Graph Prism 4.0 (Graph Pad Software Inc., San Diego, CA, USA).

## Abbreviations

LGJ: Lucerne green juice; COD: Chemical oxygen demand; CG: Crude glycerol; IN: Inorganic nutrients.

## Competing interests

The authors declare that they have no competing interests.

## Authors’ contributions

CS, FVD, OLP carried out the chemical investigations regarding the Lucerne green juice and glycerol characterization. DV contributed to the fermentation experimental work, measuring the lactic acid and glycerol content and recording the FTIR spectra, and wrote the manuscript. All authors read and approved the final manuscript.
